# Simultaneous administration of EZH2 and BET inhibitors inhibits proliferation and clonogenic ability of metastatic prostate cancer cells

**DOI:** 10.1080/14756366.2022.2163242

**Published:** 2023-01-11

**Authors:** Aide Negri, Marina Marozzi, Daniela Trisciuoglio, Dante Rotili, Antonello Mai, Federica Rizzi

**Affiliations:** a Department of Medicine and Surgery, University of Parma, Parma, Italy; b Institute of Molecular Biology and Pathology (IMBP), National Research Council (CNR) c/o Department of Biology and Biotechnology “Charles Darwin,” Sapienza University of Rome, Rome, Italy; c Department of Chemistry and Technology of Drugs, Sapienza University of Rome, Rome, Italy; d National Institute of Biostructure and Biosystems (INBB), Rome, Italy

**Keywords:** GSK126, JQ1, c-myc, metastatic prostate cancer, epigenetic drugs

## Abstract

Androgen deprivation therapy (ADT) is a common treatment for recurrent prostate cancer (PC). However, after a certain period of responsiveness, ADT resistance occurs virtually in all patients and the disease progresses to lethal metastatic castration-resistant prostate cancer (mCRPC). Aberrant expression and function of the epigenetic modifiers EZH2 and BET over activates c-myc, an oncogenic transcription factor critically contributing to mCRPC. In the present work, we tested, for the first time, the combination of an EZH2 inhibitor with a BET inhibitor in metastatic PC cells. The combination outperformed single drugs in inhibiting cell viability, cell proliferation and clonogenic ability, and concomitantly reduced both c-myc and NF-kB expression. Although these promising results will warrant further in vivo validation, they represent the first step to establishing the rationale that the proposed combination might be suitable for mCRPC treatment, by exploiting molecular targets different from androgen receptor.

## Introduction

Prostate cancer (PC) is one of the most common neoplasias among men and one of the leading causes of tumour death^1^. Currently, the clinical options for newly diagnosed PC include active surveillance, surgical removal, radiotherapy, chemotherapy and androgen deprivation therapy (ADT) or a combination of them, depending on patient’s disease stage and risk stratification^2^. Androgen deprivation therapy (ADT) constitutes a widely used approach in advanced PC. Despite recent success in developing more specific and effective ADTs, patients commonly progress to a severe form of disease, termed castration-resistant prostate cancer (CRPC) that can evolve in lethal metastatic disease (mCRPC)^2^. Although new experimental therapies such as prostate specific membrane antigen (PSMA) bound lutetium-177 and poly-ADP ribose polymerase (PARP) inhibition have shown promising results in selected cohorts of patients bearing late stage mCRPC^3^^,^^4^, the development of non-androgen receptor (AR) targeted therapies for late stage mCRPC still remains an unmet clinical need.

The aetiology of PC is complex and includes both genetic and epigenetic alterations that contribute to its onset and progression by altering the expression and function of many genes. Post-translational modifications (PTM) of histone tails have a relevant role in affecting the chromatin structure and the transcriptional landscape that leads to an unbalanced expression of oncogenes and tumour suppressor genes, finally promoting tumour growth^5–8^.

Enhancer of Zeste 2 (EZH2), the catalytic subunit of the Polycomb repressive complex 2 (PRC2), is a histone methyltransferase that contributes to the transcriptional repression of several tumour suppressor genes by the tri-methylation of lysine 27 on histone H3 (H3K27me3), an epigenetic mark positively related to chromatin compaction^9^. EZH2 expression is strongly upregulated in CRPC, particularly in neuroendocrine differentiated tumours^10^^,^^11^. However, an increasing body of evidence demonstrates that EZH2 has many "non-conventional" functions that go beyond H3K27 tri- methylation^12^. Through Polycomb repressive-independent mechanisms, EZH2 regulates the activity of several non-histone substrates that promote the transcription of genes involved in the development of AR signalling independence^13^^,^^14^. EZH2 also acts as a co-activator of critical transcription factors, including AR in CRPC cells, and sustains c-myc up-regulation in AR-negative c-myc driven PC cells^14^^,^^15^. The small molecule GSK126 selectively inhibits the EZH2 methyltransferase activity by competing with the binding of the co-substrate S-adenosyl methionine (SAM), which is the methyl donor of the methyltransferase reaction^16^. GSK126 induces apoptosis and re-expression of tumour suppressor genes in many cancer cell lines and inhibits tumour growth in animal models^17^. EZH2 ablation was effective to reduce cell growth and migration in many different PC cell lines^18^. However, EZH2 inhibitors (EZH2i), including GSK126 were minimally effective when administered as single agents in CRPC cells, while the combination with chemotherapy or radiotherapy is a feasible approach to enhance their efficacy^19^^,^^20^.

By using histone PTM global profiling, it has been demonstrated that EZH2 inhibition induces a global change of the epigenetic signature of cancer cells and yields, concomitantly, H3K27me3 loss and H3K27ac gain^21^. In many solid tumour cell lines, the combination of EZH2i with the inhibition of acetylated histone readers suppresses the H3K27me:H3K27ac crosstalk and re-sensitize cancer cells to EZH2i^21^. Thus, the combination of EZH2i with other epigenetic drugs capable of disrupting the transcriptional reprogramming of cancer cells triggered by EZH2-targeted therapy may be a rational strategy to increase EZH2 inhibition effectiveness, also in advanced mCRPC.

Bromodomain-Containing Protein 4 (BRD4) is a member of the BET family of chromatin readers that is markedly overexpressed in CRPC^22–24^; moreover, increased levels of BRD4 are associated with a poor prognosis^25^. BRD4 recruits the positive transcription elongation factor P-TEFb and other transcription factors involved in the transcription of the oncogene c-myc^26^. JQ1 is a pan-BET inhibitor (BETi) with a high affinity for BRD4^27^ whose anti-tumour activity has been proved in different cancer cell types, including PC cells, and murine models^22^^,^^28^.

c-myc is a downstream mediator of AR signalling, however, it can also drive cell growth in the absence of AR ligand^29^. Consistently, amplification of c-myc is among the most common genetic alterations observed in CRPC, where it plays a central role by dysregulating cell division and cell cycle, promoting metabolic adaptation, and finally contributing to cell survival^30^. For the above-mentioned characteristics, c-myc represents one of the most promising AR-independent targets to develop CRPC-effective therapies. Unfortunately, until now c-myc has proven to be an “undruggable” target^30^^,^^31^.

Since both EZH2 and BRD4 are overexpressed in CRPC cells^11^^,^^22^ and contribute, by different mechanisms, to c-myc up-regulation^32^^,^^33^, we decided to test whether the combination of EZH2i and BETi may elicit a synergistic cytotoxic/cytostatic effect on CRPC cells, possibly through c-myc levels reduction. CRPC is a very heterogeneous disease state characterised by lack of AR activity. This heterogeneity challenges standard approaches to CRPC management and highlights the need for personalised cancer therapy^34^.

For this purpose, we tested the efficacy of GSK126 and JQ1 simultaneous administration in DU145 and PC3 cells, which are perhaps the two most characterised and widely used androgen-independent and metastatic PC cell lines that mimic the most advanced stages of the disease^35^. LNCaP cells, originally isolated as androgen-dependent cells^35^, were also included in the first part of the experimental design. First, we tested the effects of the combination on cell viability, cell proliferation, and clonogenic ability of the selected cell lines. Then, we measured how GSK126 and JQ1, given as single agents or in combination, affect the expression of EZH2, BRD4, H3K27me3, c-myc, and NF-kB. Finally, we measured the effects of the combination on cell cycle progression and investigated the mechanisms of cell death, focussing our attention on caspases activation and apoptosis induction.

## Materials and methods

### Reagents

GSK126 and JQ1 were gently provided by Dr. Dante Rotili (Department of Drug, Chemistry, and Technologies, “Sapienza” University of Rome, Italy). Both compounds were dissolved in DMSO at the concentration of 100 mM, aliquoted, and stored at −20 °C until use.

### Cell growth conditions

DU145, PC3, and LNCaP cell lines were purchased from the American Type Culture Collection (ATCC, Manassas, VA, USA). DU145 cells and PC3 cells were used at passages between 50 and 55. LnCaP cells were used at passages between 30 and 32. All the cell lines were cultured in RPMI 1640 (Corning, Mediatech, VA 20109-USA) supplemented with 10% of foetal bovine serum, 100 U/mL penicillin, 100 µg/mL streptomycin, and 2 mM L-glutamine (Gibco, Life Technologies, Carlsbad, CA, USA). Cells were incubated at 37 °C under a 5% CO_2_ humidified atmosphere. When cells reached 70–80% confluence, cell medium was aspirated, the cells were washed twice with 1X PBS and detached from the cell culture plate with trypsin/EDTA 0.5 g/mL, at 37 C° for 3 min. Trypsin/EDTA was inhibited by medium addition. Detached cells were pelleted by centrifugation, opportunely diluted in cell medium, stained with Trypan Blue and manually counted by mean of a Bürker chamber.

### Cell viability assay

DU145 and PC3 cell lines were seeded in a 96-well plate (white view plate-96 TC Perkin Elmer, Waltham, MA, USA) at the density of 8000 cells/well and treated 24 h later with increasing concentrations of GSK126 or JQ1 (1 µM, 25 µM, 50 µM, 75 µM, 100 µM, and 200 µM). A final concentration of 0.03% DMSO was added to control cells (CTR). After 48 h of treatment, the Luminescence ATP Detection Assay System (ATPlite, PerkinElmer) was carried out to measure cell viability according to the vendor’s directions. Briefly, 50 μL of mammalian cell lysis solution were added to each well and the plate was shaken for 5 min. 50 μL of substrate solution were dispensed to each well and the plate was shaken again for 5 min and incubated in the dark for 10 min. The emitted luminescence was measured using the EnSpire® plate reader (PerkinElmer). For each drug, a dose-response curve was generated and the IC50 (the half-maximal inhibitory concentration) was calculated using non-linear regression analysis (four parameters logistic curve), via SigmaPlot software (version 12.0).

ATPlite assay was also carried out in DU145 and PC3 to evaluate the combinatorial effect of GSK126 and JQ1 on cell viability. Cells were seeded in a 96-well plate (white view plate-96 TC Perkin Elmer) at the density of 8000 cells/well and 24 h later were treated with GSK126 at the established IC50 in the absence or presence of increasing concentrations of JQ1 (0.1 μM, 1 μM, 10 μM, 20 μM, 30 μM and 50 μM). A final concentration of 0.03% DMSO was added to CTR. After 48 h of treatment, the ATPlite assay was performed as previously described.

### Drugs combination index calculation

DU145, PC3 and LNCaP cell lines were seeded in a 96-well plate (white view plate-96 TC Perkin Elmer) at the density of 10 000 cells/well and 24 h later were treated with increasing concentration of GSK126 (10 µM, 20 µM, 30 µM, and 40 µM for DU145; 6 µM, 14 µM, 20 µM and 28 µM for PC3 and LNCaP) and/or JQ1 (0.3 µM, 0.7 µM, 1 µM and 1.4 µM for all cell lines). After 48 h of treatment, the ATPlite assay was per-formed as previously described. Dose-effect analysis was completed in accordance with the Chou Talalay method using CalcuSyn Software (CalcuSyn software, Biosoft, Cambridge). The combination index (CI) was automatically generated by the software over a range of fractionated affected levels at different growth inhibition percentages. CI values of <1, =1, and >1 indicate synergistic, additive, and antagonistic effects, respectively.

### Crystal violet assay

DU145 and PC3 were seeded at the density of 170 000 cells/well in a six-well plate and 24 h later were treated with GSK126 and/or JQ1. After 48 h of treatment, cell proliferation was evaluated using the Crystal Violet assay. Cells were washed in PBS 1X, fixed in 3% formaldehyde, and stained with Crystal Violet reagent (Applichem, Darmstadt, Germany) at room temperature (RT). After two washes in distilled water, photos were taken of the stained cells. Finally, the Crystal Violet reagent was extracted with a 0.1 M sodium citrate solution and transferred to a 96-well plate. Absorbance was read at 540 nm using the EnSpire® plate reader (PerkinElmer).

### Clonogenic assay

DU145 and PC3 were seeded at the density of 1000 cells/dish in 35 mm x 10 mm dishes and 24 h later were treated with GSK126 1 µM and/or JQ1 0.033 µM. The treatment was repeated every 3 days. After 9 days of treatment, colony formation was evaluated using Crystal Violet staining. Cells were washed in PBS 1X, fixed in 3% formaldehyde and stained with Crystal Violet reagent (Applichem, Darmstadt, Germany) at room temperature (RT). After two washes in distilled water, photos of the stained cells were taken. The area occupied by the colonies was quantified by an automated single-color image counting, using ImageJ software (https://imagej.nih.gov/ij/download.html), and reported in the graph as the percentage of the area measured in the control untreated sample.

### Live/dead assay

DU145 and PC3 were seeded at the density of 170 000 cells/well in a six-well plate and 24 h later were treated with GSK126 and/or JQ1. After 48 h and 72 h of treatment, cell death was evaluated using the LIVE/DEAD Fixable Dead Cell Stain Kit (Invitrogen, Waltham, Massachusetts, USA). Cells were collected, washed, and suspended in PBS 1X at the density of 1 × 106 cells/mL, then 0.5 µL of the green fluorescent solution was added to each cell suspension. After 30 min of incubation in the dark at room temperature (RT), the flu-orescence emission was measured at 530 nm using the Invitrogen TMAttune TM NxT Flow Cytometer (Invitrogen). Data were analysed with FlowJo TM version 10 software (BD Biosciences CA, USA).

### Annexin V/PI staining

DU145 and PC3 were seeded at the density of 170 000 cells/well in a six-well plate and 24 h later were treated with GSK126 and/or JQ1. After 48 h and 72 h of treatment, apoptosis was evaluated using the FITC Annexin V/Dead cell Apoptosis kit (Invitrogen). Cells were collected, washed with PBS 1X, suspended in 200 µL of 1X-Annexin-binding buffer, and incubated with 5 µL of FITC Annexin V and 1 µL of Propidium iodide 100 µg/mL for 15 min at RT, in the dark. After the incubation period, 400 µL of 1X Annexin V-binding buffer was added. The cells were analysed by the CytoFlex flow cytometer (Beckman Coulter, Fullerton, CA, USA), measuring the fluorescence emission at 530 nm. Data were analysed with, Expo ADC software (Beckman Coulter).

### Cell cycle analysis

DU145 and PC3 were seeded at the density of 170 000 cells/well in a six-well plate and 24 h later were treated with GSK126 and/or JQ1. After 48 h and 72 h of treatment, cells distribution in cell cycle phases was evaluated using the FxCycle™ PI/RNase Staining Solution kit (Thermo Fisher Scientific, Waltham, Massachusetts, USA). Cells were collected, washed with PBS 1X, fixed in 70% ethanol for 1 h at −20 °C, and washed again in PBS 1X. 0.5 mL of FxCycle™ PI/RNase Staining Solution stain was added to cellular pellets and incubated in the dark at RT for 30 min. Then, cellular suspensions were analysed with the Attune NxT Flow Cytometer (Invitrogen) measuring the fluorescence emission at 585/42.

### Caspase 3/7 activity

DU145 and PC3 cell lines were seeded in a 96-well plate (white view plate-96 TC Per-kin Elmer) at the density of 10 000 cells/well and treated 24 h later with GSK126 and/or JQ1. After 48 h and 72 h of treatment, caspase 3/7 activity was measured with the Caspase-Glo® 3/7 assay kit (Promega, Madison, WI, USA); 100 µL of the Caspase-Glo® 3/7 reagent were added to each well and cells were incubated for 30 min at room temperature. The emitted luminescence was measured using the EnSpire® plate reader (PerkinElmer).

### Protein extraction, SDS-Page, and Western Blot analysis

DU145 and PC3 were seeded at the density of 170 000 cells/well in a six-well plate and 24 h later were treated with GSK126 and/or JQ1. After 48 h and 72 h of treatment, 75 μL of RIPA lysis buffer (50 mM TRIS-HCL pH 7.4 100 mM NaCl, 1% Triton-X-100) supplemented with phosphatase and protease inhibitor cocktails (Sigma-Aldrich, Saint-Louis, MO, USA), were added to each well. Cells were gently scraped, collected, and maintained un-der slow rotation for 45 min at 4 °C. Protein concentration was measured using the Bio-Rad DC Protein Assay (Bio-Rad, Hercules, CA, USA) according to the manufacturer’s protocol. Absorbance at 750 nm was measured by the EnSpire® plate reader (PerkinElmer). Equal amounts of proteins were loaded on a 12% polyacrylamide gel (SDS-PAGE) and transferred onto a polyvinylidene difluoride (PVDF) membrane (Millipore, Billerica, MA, USA). Membranes were incubated with the following primary antibodies overnight at 4 °C: rabbit polyclonal anti-c-Myc (#9402 Cell Signalling Technology, Denver, MA, USA), dilution 1:1000, rabbit polyclonal anti-H3K27me3 (07–449, Millipore), dilution 1:200, mouse monoclonal anti-NF-κB (#6956 Cell Signalling Technology), dilution 1:1000, mouse monoclonal anti-PARP (sc8007 Santa Cruz, CA, USA), dilution 1:500, rabbit polyclonal anti-cPARP (#9541 Cell Signalling Technology), dilution 1:250, rabbit polyclonal anti-BRD4 (#13440 Cell Signalling Technology), dilution 1:250, rabbit polyclonal anti-EZH2 (07–689, Millipore), dilution 1:1000, rabbit monoclonal anti-p21 (#2947 Cell Signalling Technology), dilution 1:1000, mouse monoclonal anti-β-actin (SC81178 Santa-Cruz Biotechnology, Dallas, TX, USA) dilution 1:500. Membranes were hybridised with the following secondary antibody conjugated with horseradish peroxidase: goat anti-mouse IgG (dilution 1:5000) or a goat anti-rabbit IgG (dilution 1:150 000, 1:50 000, 1:20 000) (Sigma-Aldrich) and immunoreactive bands were detected by the BM chemiluminescence Blotting Substrate, POD (Roche, Basel, CH) and exposed to Hyperfilm ECL photographic films (GE Healthcare, Chalfont St. Giles, Bucks, UK). Densitometric analysis of each band was performed with the Quantity One® software (Bio-Rad).

### Statistical analysis

Statistical analysis was performed with the IBM SPSS statistical package software, version 27 (International Business Machines Corporation, Armonk, NY, USA). The statistical difference in the means of the experimental groups was tested by ANOVA, followed by *post hoc* multiple comparisons (multiple t-test corrected for Bonferroni). Differences were considered significant if the Bonferroni-adjusted *p* values was less than or equal to 0.05 at the 95% C.I. All the *p* values reported in the figure captions are actually Bonferroni-adjusted *p* values. Graphs were generated by using the GraphPad Prism software, version 8 (GraphPad Software, San Diego, CA, USA).

## Results

### Effects of GSK126 and JQ1 combined treatment on cell viability, proliferation, and clonogenic ability

**Figure 1.**
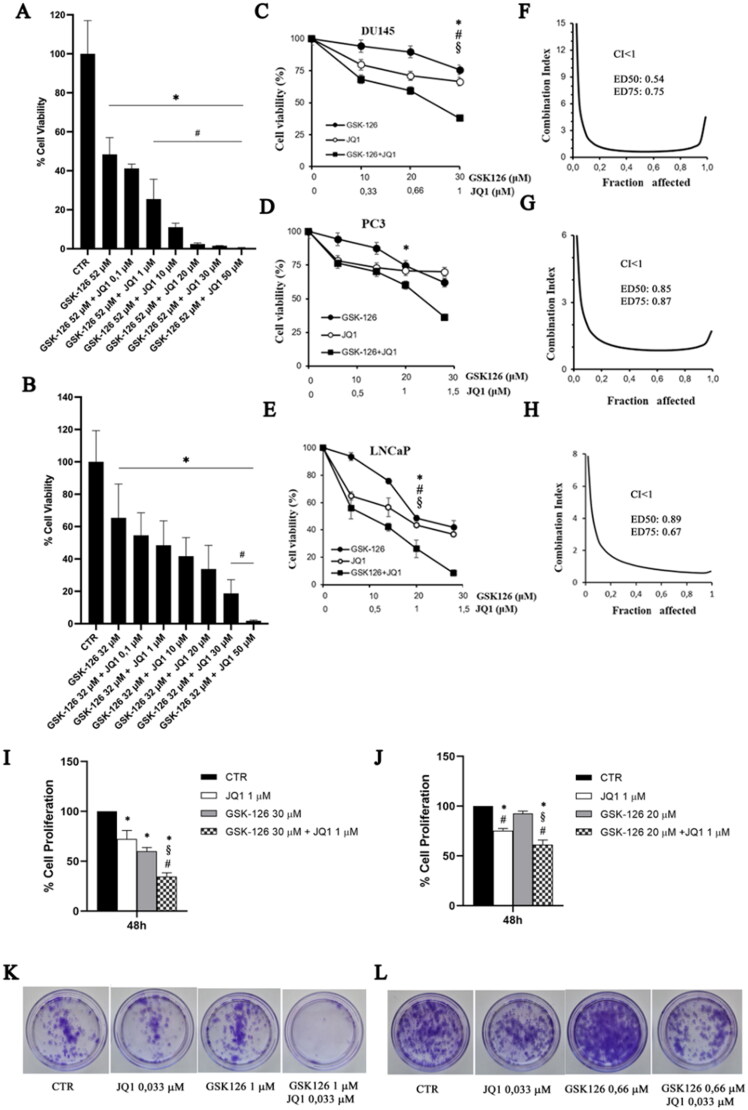
GSK126 and JQ1 combined treatment reduces cell viability, cell proliferation, and clonogenic ability of metastatic PC cells. DU145 (A) and PC3 (B) cell lines were treated for 48h with GSK126 IC_50_ doses in the absence or presence of increasing concentrations of JQ1 as indicated. Cell viability was determined by the ATPlite assay and expressed as percentage of the control. Data represent the mean ± SEM of three independent experiments. (C–E) Analysis of cell viability in DU145, PC3, and LNCaP cells treated with GSK126 and JQ1 alone or in combination (at the fixed concentration ratio of 30:1 for DU145 and 20:1 for PC3 and LNCaP) for 48h. The results are reported as percentage of the control and represent the mean ± SD of the two independent experiments performed in triplicate. *Adjusted *p* < 0.05 indicates significant differences vs. control (CTR); § adjusted *p* < 0.05 indicates significant differences vs. JQ1; # adjusted *p* < 0.05 indicates significant differences vs. GSK126 (One-way ANOVA followed by multiple t-test with Bonferroni correction). (F–H) Calculation of the combination index (CI). The results represent the mean of two independent experiments performed in triplicate. On the y axis is represented the combination index, on the x axis the fraction of cells inhibited. CI values of <1, =1, and >1 indicate synergistic, additive, and antagonistic effects, respectively. DU145 (I) and PC3 (J) cell proliferation was determined by crystal violet assay after 48h of treatment with GSK126 or JQ1 and with the two drugs in combination at the concentrations reported in the figure (fixed concentration ratio of 30:1 for DU145 and 20:1 for PC3). DU145 (K) and PC3 (L) colony formation ability was determined by clonogenic assay after 9 days of treatment with GSK126 and JQ1 alone or in combination at the concentrations reported in the figure (fixed concentration ratio of 30:1 for DU145 and 20:1 for PC3). Images are representative of two independent experiments.

We calculated the IC50 values of GSK126 and JQ1 by treating DU145 and PC3 cells with increasing concentrations of the two drugs (tested concentration range: 0–200 µM). After 48 h both drugs reduced cell viability in a dose-dependent manner (Figure S1). The calculated IC50 value for GSK126 was 52 µM in DU145 cells (Figure S1A) and 32 µM in PC3 cells (Figure S1C). JQ1 IC50 was 51 µM in DU145 (Figure S1B) and 45 µM in PC3 (Figure S1D). Then, we treated DU145 (Figure 1(A)) and PC3 (Figure 1(B)) with GSK126 IC50 doses and increasing concentration of JQ1 (range 0–50 µM) and evaluated the effects of the combined treatment on cell viability after 48 h. The combination of GSK126 with doses of JQ1 as little as 1 µM significantly reduced the viability of DU145 compared to both untreated and single drug-treated cells (Figure 1(A)), while in PC3 cells, a positive effect of the combination over the single-agent treatment became appreciable with doses of JQ1 higher than 20 µM (Figure 1(B)). To evaluate whether GSK126 and JQ1 acted in an additive or synergistic way to reduce cell viability, combination studies were performed using a fixed-ratio experimental design. Taking into account that the calculated IC50 for GSK126 was higher in DU145 than in PC3 cells, we fixed the GSK126:JQ1 ratio at the values of 30:1 for DU145 and 20:1 for PC3 cells. As shown by growth inhibition curves (Figure 1(C,D)) and analysis of drug interactions (Figure 1(F,G)) simultaneous treatment with GSK126 and JQ1 for 48 h resulted in a synergistic effect, being for DU145 cells CI at ED50:0.54 and CI at ED75: 0.75, whilst for PC3 cells CI at ED50:0.83 and CI at ED75: 0.87. The combined treatment also reduced cell viability and had a synergistic effect on LNCaP cells CI at ED50:0.89 and CI at ED75:0.67 (Figure 1(E–H)). However, taking into account that these cells mimic an earlier stage of disease according to their classification as androgen-dependent and AR-positive cells^35^, we focussed on DU145 and PC3 cells for the rest of experiments.

Based on the results of the combination index analysis that showed a synergistic effect of the two drugs on cell viability reduction when used at the concentration ratio GSK126:JQ1 of 30:1 for DU145 and 20:1 for PC3, we fixed these con-centration ratios for all the subsequent experiments.

We performed a crystal violet assay to evaluate the effects of the drug combination on cell proliferation. We treated both cell lines with sub-IC50 doses of GSK126 (30 µM for DU145 and 20 µM for PC3), 1 µM of JQ1, or the combination of both drugs, as indicated in the figure legends (Figure 1(I,J)). The treatment with GSK126 or JQ1 reduced DU145 cell proliferation compared to control untreated cells, however, the combined treatment resulted in a more marked decrease in cell proliferation compared to both control and single-drug treatments (Figure 1(I)). In PC3 cells, the combined treatment was still more effective than each single drug to reduce cell proliferation (Figure 1(J)); however, the effect was smaller than that observed in DU145 cells.

In good agreement with cell viability and cell proliferation assays, we found that the combined treatment dramatically suppressed colony formation in DU145 cells (Figure 1(K), quantification in Supplementary Figure 2A) and to a less extent in PC3 cells (Figure 1(L), quantification in Supplementary Figure 2B), while single-drug treatments had a very low or null effect compared to control (Figure 1(K,L)). As for the other experiments, we kept the ratio GSK126:JQ1 fixed at 30:1 for DU145 and 20:1 for PC3 also for the clonogenic assay. Therefore, by keeping in mind that the slope of the GSK126 IC50 curve was larger in PC3 than in DU145 cells (Figure S1A), we cannot exclude that having treated PC3 cells with lower concentrations of GSK126 than those used to treat DU145 cells, negatively and a nonlinearly affected the inhibition of cell proliferation.

### Effects of GSK126 and JQ1 combined treatment on the expression of EZH2, BRD4, and their molecular targets

**Figure 2.**
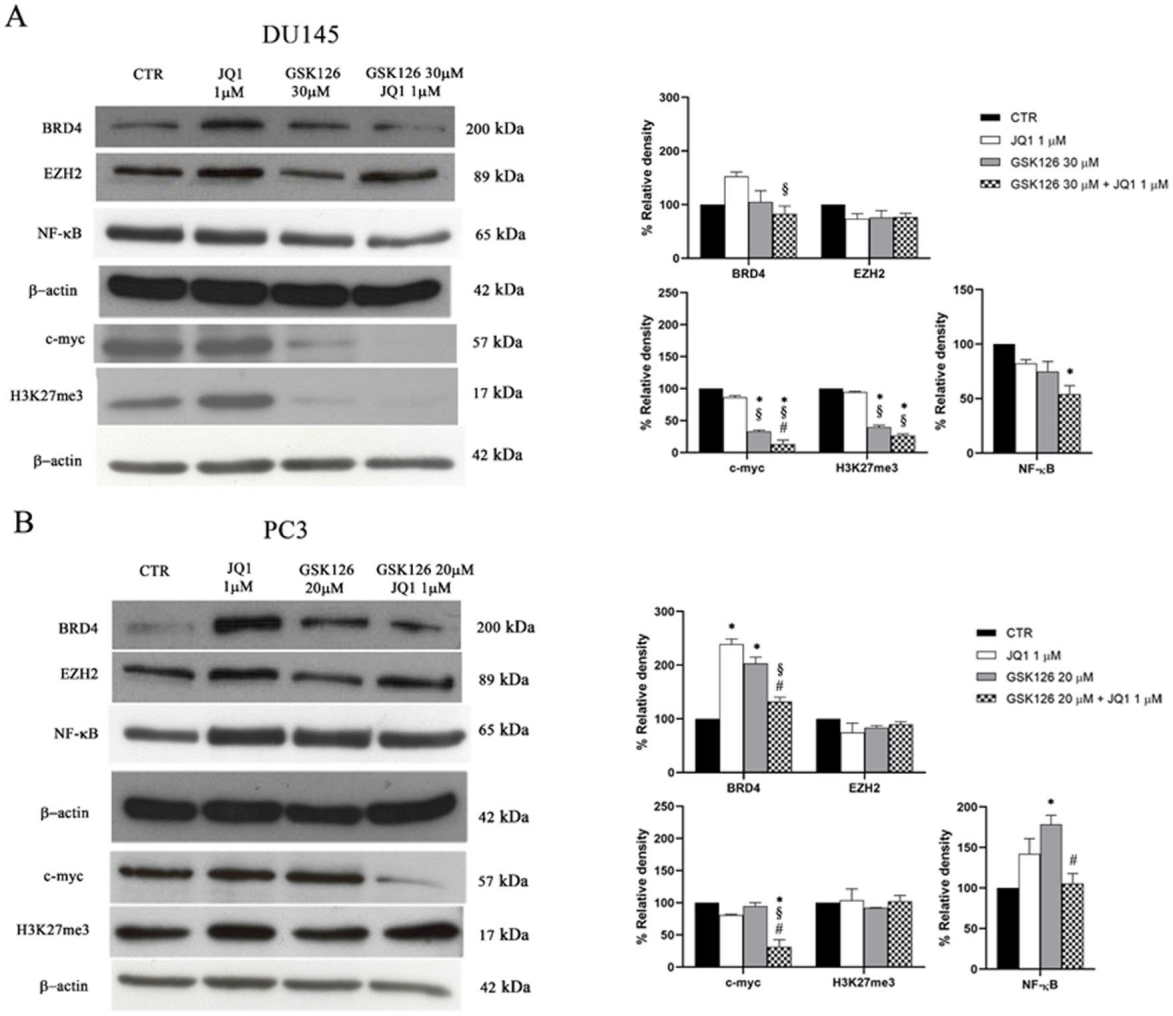
Effects of GSK126 and JQ1 combined treatment on EZH2, BRD4, and their molecular targets. BRD4, EZH2, NF-kB, c-myc, and H3K27me3 protein expression was evaluated by western blot analysis in DU145 (A) and PC3 (B) after 72h of treatment with GSK126 or JQ1 and with the two drugs in combination as indicated. β-actin was used as the loading control. Immunoreactive bands were quantified by densitometric analysis and normalised to β-actin signal. The normalised densitometric data reported in the bar graphs are expressed as percentage of the control and represent the mean ± SEM of three independent experiments. *Adjusted *p* < 0.05 indicates significant differences vs. CTR; § adjusted *p* < 0.05 indicates significant differences vs. JQ1; # adjusted *p* < 0.05 indicates significant differences vs. GSK126 (one-way ANOVA followed by multiple t-test with Bonferroni correction).

We evaluated the effects of GSK126, JQ1, or their combination on the expression of EZH2 and BRD4 in both DU145 and PC3 cells (Figure 2). EZH2 levels were not affected by JQ1, GSK126, or by the combination of the two compounds (Figure 2(A,B)). Differently, the expression of BRD4 increased both in DU145 cells (Figure 2(A)) and more significantly in PC3 cells (Figure 2(B)) following JQ1 treatment. This effect was abolished (in DU145 cells) or drastically reduced (in PC3 cells) when JQ1 was combined with GSK126 (Figure 2(A,B)).

Then, we evaluated the effects of the treatments on the expression level of c-myc. In line with previous observations JQ1, given as a single drug at 1 µM concentration, did not affect c-myc protein levels in AR-negative PC cells that are almost unresponsive to BET inhibition (Figure 2(A,B))^36^. By treating both cell lines with increasing doses of JQ1, we observed a reduction of c-myc levels compared to controls only in PC3 cells treated with 50 µM JQ1 (Supplementary Figure 3A–B). Differently, GSK126 reduced c-myc levels both when used as a single drug and in combination with JQ1 in DU145, and only when combined with JQ1 in PC3 cells (Figure 2(A,B)). Of note, the combined treatment outperformed both GSK126 and JQ1 single treatment in reducing c-myc levels in both cell lines (Figure 2(A,B)) and was paralleled by an increased reduction of H3K27me3 only in DU145 cells (Figure 2(A)). This result suggests that EZH2 contributes to c-myc up-regulation through a Polycomb repressive-independent mechanism that requires the methylation of a substrate different from H3K27^14^.

It has been demonstrated that genetic ablation or treatment with EZH2i leads to feedback activation of NF-κB signalling in PC cells^37^. This mechanism has been proposed as one of the adaptive responses of PC cells to EZH2 ablation that in turn may result in the acquired resistance to EZH2i drugs^37^. For this reason, we tested the effects of JQ1, GSK126, and their combination on the expression of NF-kB. We found that GSK126 significantly increased NF-kB levels in PC3 cells (Figure 2(A,B)). Interestingly, the combined treatment returned NF-kB levels back to control values in this cell line (Figure 2(B)), and to levels lower than control in DU145 cells (Figure 2(A)).

### Effects of GSK126 and JQ1 combined treatment on cell death, apoptosis, and cell cycle progression

**Figure 3.**
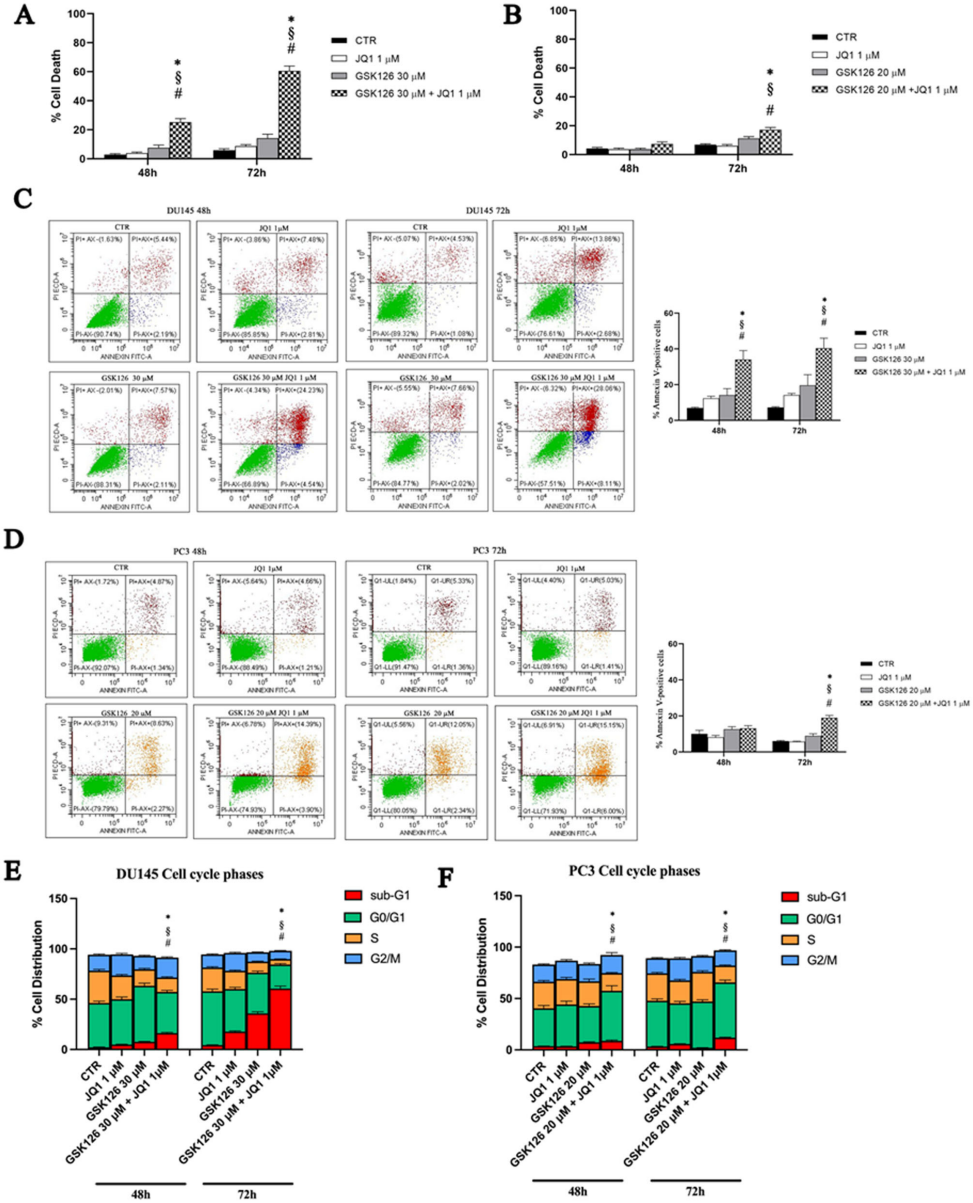
Effects of GSK126 and JQ1 combined treatment on cell death, apoptosis and cell cycle progression. After 48 h and 72 h of treatment with GSK126 or JQ1 and with the two drugs in combination at the concentration indicated in the figure, cell dead was determined by Live/Dead assay in DU145 (A) and PC3 (B) and reported in the bar graph as percentage of the control. Apoptosis was determined by Annexin V/PI double staining in DU145 (C) and PC3 (D) and reported in the bar graph as percentage of the control. On the Y-axis is reported the sum of Annexin V-positive cells plus Annexin V/PI positive cells. The percentage of cells distribution in the cell cycle phases was determined in DU145 (E) and PC3 (F) by FxCycle™ PI/Rnase staining. Data represent the mean ± SEM of three independent experiments. *Adjusted *p* < 0.05 indicates significant difference vs. CTR; § adjusted *p* < 0.05 indicates significant difference vs. JQ1; # adjusted *p* < 0.05 indicates significant difference vs. GSK126 (one-way ANOVA followed by multiple t-test with Bonferroni correction).

We evaluated the effects of EZH2i and BETi combination on cell death, apoptosis, and cell cycle progression (Figure 3). The percentage of cell death was determined by using a fluorimetric Live/Dead assay. In DU145 cells, the combined treatment produced a significant increase in cell death compared to both control and single drug-treated samples, already after 48 h (Figure 3(A)). In PC3 cells, a significant effect was appreciable after 72 h of treatment (Figure 3(B)). In both cell lines, single drug treatments did not induce a significant increase in cell death compared to untreated controls (Figure 3(A,B)). Apoptosis induction was evaluated by the Annexin V/PI double staining analysis (Figure 3(C,D)). In DU145 cells receiving the combined treatment, the percentage of cellular apoptosis significantly increased compared to both control and single drug-treated cells, regardless of the treatment time (Figure 3(C)). Differently, in PC3 cells, the drug combination yielded a very moderate increase of Annexin V-positive cells only after 72 h of treatment (Figure 3(D)), while single drug treatments were ineffective (Figure 3(D)). We then evaluated the effects EZH2i and BETi combination on the cell cycle (Figure 3(E,F)). We observed a strong time-dependent increase of the sum of the sub-G1 and G0/G1 phases, concomitantly with a reduction of the S, G2 phases in DU145 cells treated with the drug combination, compared to both control and single drug-treated cells (Figure 3(E)). The sub-G1 peak is suggestive of the accumulation of apoptotic cells that exit the cell cycle after a G0/G1 arrest. In PC3 cells, we observed an increase in the G1 phase and a time-dependent reduction of the S and G2/M phases following the combined treatment compared to both control and single agent-treated cells (Figure 3(F)) which is suggestive of G0/G1 phase cell cycle block.

In these cells, the sub-G1 peak was barely detectable after 72 h of treatment (Figure 3(F)). Following 72 h treatment with the combination of the two drugs, the expression of cyclin D1 is significantly reduced compared to control and each single drug treatment in DU145, and to control and GSK126 treatment in PC3 cells (data not shown).

### Effects of GSK126 and JQ1 combined treatment induced cell death by caspases independent mechanisms in DU145 and PC3 cells

**Figure 4.**
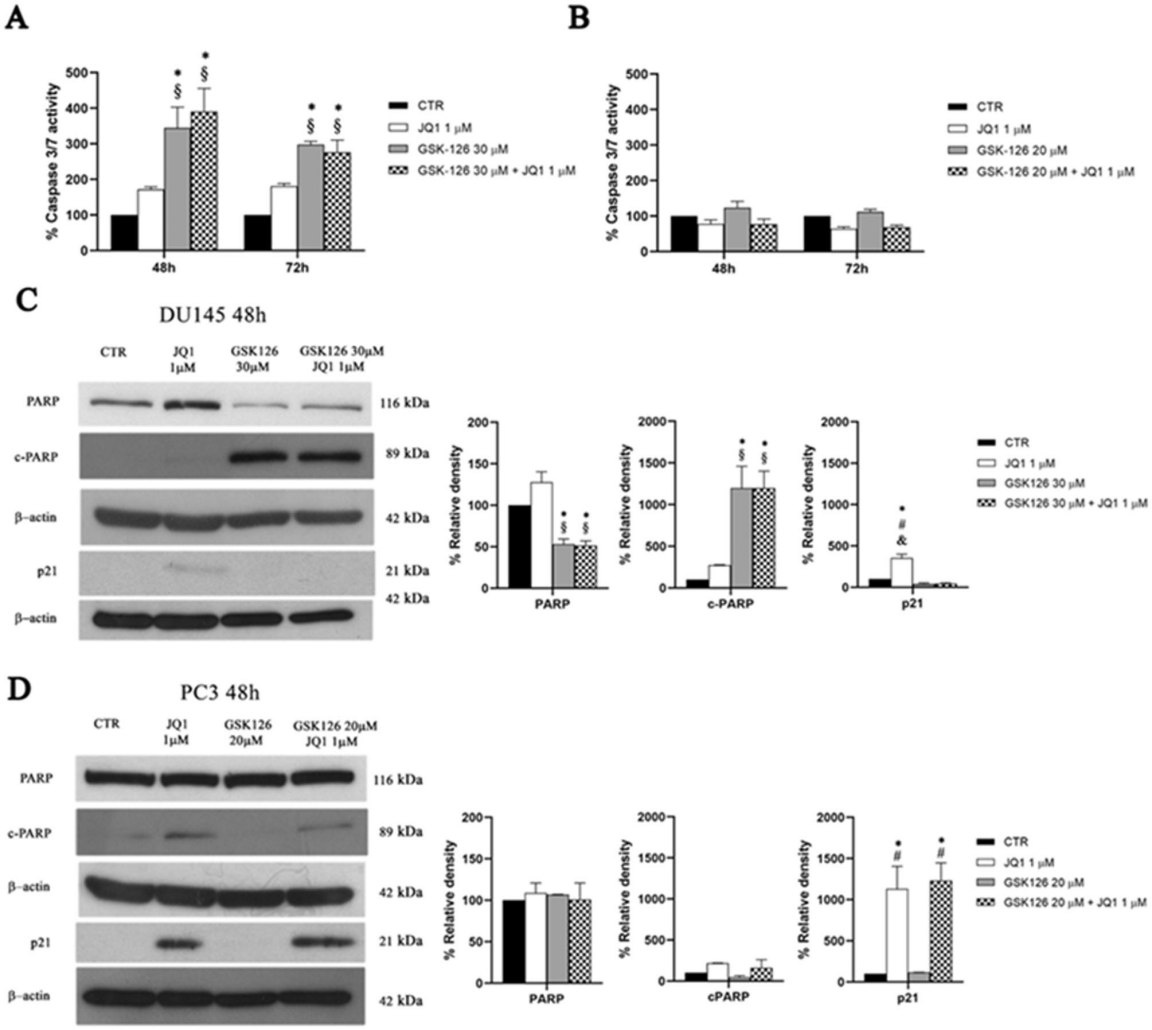
Analysis of cell death mechanisms induced by GSK126 and JQ1 combined treatments. Percentage of DU145 (A) and PC3 (B) caspase3/7 activity was determined by Caspase-Glo® 3/7 assay after 48 h and 72 h of treatment GSK126 or JQ1 and with the two drugs in combination as indicated. The measure of the enzymatic activity measured after the treatments has been reported in the bar graphs as percentage of the control. Data are the mean ± SEM of three independent experiments. *Adjusted *p* < 0.05 indicates significant difference vs. CTR; § adjusted *p* < 0.05 indicates significant difference vs. JQ1 (one-way ANOVA followed by multiple t-test with Bonferroni correction). PARP, cPARP, and p21 protein expression was evaluated by western blot analysis in DU145 (C) and PC3 (D) after 48h of treatment with GSK126 or JQ1 and with the two drugs in combination as indicated. β-actin was used as the loading control. The expression levels of PARP, cPARP, and p21 was measured by densitometric analysis and normalised to β-actin. The normalised densitometric data reported in the bar graphs are expressed as percentage of the control and represent the mean ± SEM of three independent experiments. * Adjusted *p* < 0.05 indicates significant differences vs. CTR; § adjusted p < 0.05 indicates significant differences vs. JQ1; # adjusted *p* < 0.05 indicates significant differences vs. GSK126 (one-way ANOVA followed by multiple t-test with Bonferroni correction).

The results of the cell cycle analysis and Annexin V staining indicated that the combined treatment produced different outcomes in DU145 and PC3 cells, being more clearly cytotoxic in the former and cytostatic in the latter. Therefore, we supposed that the drug combination might act through different molecular mechanisms in the two cell lines. First, we measured the activity of effector caspases 3/7 both after single drug- and combined-treatment (Figure 4(A,B)).

In DU145 cells, GSK126 induced caspase 3/7 activation to a similar extent both when used as a single drug and in combination with JQ1 (Figure 4(A)). Caspases activation was accompanied by a significant increase of cleaved PARP (cPARP) and the reduction of total PARP levels, without significant effects on p21 expression, both following GSK126 and the combined treatment (Figure 4(C)). JQ1 single treatment slightly increased p21 levels without effects on caspases activation and PARP cleavage (Figure 4(C)).

Differently, in PC3 cells, neither the combination nor the single drug treatments yielded caspases 3/7 activation (Figure 4(B)). In these cells, JQ1 slightly increased cPARP concomitantly with a dramatic increase of p21 levels, without effects on total PARP both when used as a single drug and in combination with GSK126 (Figure 4(D)).

Altogether, cell death, Annexin V, caspases and PARP/cPARP data support the idea that the combined treatment outperforms the cytotoxic effect of each single drug and that caspases-independent mechanisms contributed to programmed cell death induction.

## Discussion and conclusions

The current standard-of-care of systemic treatment for metastatic hormone sensitive prostate cancer includes ADT combined with chemotherapy and advanced androgen-targeted therapy^2^. Although these therapeutic protocols have been proven effective to delay progression to mCRPC, at least for patients fit enough to withstand the increased adverse effects of the combinatorial protocols^38^, they were significantly correlated to an increase of AR-negative mCRPC that are finally incurable^39^^,^^40^. Therefore, the development of non-androgen receptor (AR) targeted therapies for late stage mCRPC, still remains an active field of research. Aberrant expression and function of EZH2 and BET are, among all the epigenetic alterations observed in PC, those that maximally contribute to the development of AR-indifference^34^. However, the administration of EZHi and BETi as mono-therapy has not achieved successful results in the treatment of CRPC^41^^,^^42^.

In the present work, we showed for the first time that the simultaneous inhibition of EZH2 and BET is effective to inhibit the proliferation of metastatic PC cell lines mimicking different stages of the human disease. This is particularly relevant for DU145 and PC3 cells that are both EZH2i- and BETi-insensitive cell lines^36^^,^^42^^,^^43^.

In agreement with these published results, both cell lines responded to single agent treatment only at high doses, as we established following IC_50_ calculation for both GSK126 and JQ1.

We then evaluated the effects of the two drugs in combination by treating each cell line with the IC_50_ dose of GSK126 and increasing doses of JQ1. The cell viability of cells receiving the drug combination was significantly reduced compared to that of cells receiving single-agent treatments. The analysis of the pharmacological interaction between GSK126 and JQ1 revealed the existence of a synergistic action when the two drugs were used in the fixed ratio (GSK126:JQ1) of 30:1 for DU145 and of 20:1 for PC3 cells.

Nor the combined treatment or the administration of the two single drugs altered EZH2 expression. Differently, JQ1 produced the upregulation of BRD4 in both cell lines. This feedback effect was counteracted by the simultaneous administration of GSK126. In addition, GSK126 reduced H3K27me3 levels only in DU145 cells, when used both as a single drug and in combination. These results were not unexpected, considering that in CRPC cells the oncogenic activity of EZH2 is largely Polycomb-independent, and therefore not strictly dependent on H3K27me3 levels^13^^,^^14^.

Our results showed that the treatment of mCRPC cells with GSK126 and JQ1 combination dramatically reduced c-myc levels outperforming the result yielded by single drug treatments. Indeed, the treatment with 1 µM JQ1 did not affect c-myc expression at all, both in DU145 and PC3 cells that are considered, among all the CRPC cells, the most resistant to BETi^36^. Since c-myc amplification and overexpression is frequent in CRPC, it is reasonable to believe that the effects on cell proliferation, cell death, and cell cycle progression elicited in DU145 and PC3 cells by the combined treatment are mediated, at least in part, by its downregulation^26^^,^^36^. Interestingly, in DU145 cells, GSK126 moderately reduced c-myc levels when used as a single treatment, suggesting that EZH2 contributed to up-regulate c-myc levels through a methylation-dependent mechanism^13^^,^^14^. However, in line with previous observations, c-myc inhibition alone had a limited effect on cell viability, and required the simultaneous inhibition of BET activity to yield a significant effect on cell death induction^36^.

We found that the administration of GSK126 significantly increased the expression of the p65 subunit of NF-kB in PC3 cells. The co-administration of JQ1 counteracted the upregulation of p65 elicited by GSK126 in PC3 cells and significantly reduced p65 levels in DU145 cells compared to control. These findings are relevant because feedback activation of NF-kB is one of the adaptive responses of PC cells to EZH2 inhibition that contributes to the acquired resistance to EZH2i drugs^37^.

Interestingly, c-myc is a well-known transcriptional target of NF-κB^44^, and BRD4 recruits Mediators and NF-κB’s p65 subunit to form transcriptional super-enhancers that regulate the transcription of oncogenes in various cancers^45^. The combination of EZH2i and BETi may be advantageous to inhibit the transcriptional activation of c-myc and other master regulators of transcription that are relevant for AR-independent growth^46^. BRD4 binds to hyperacetylated p65 and activates NF-κB in many different type of cancer cells^47^. Thus, blocking the interaction between BRD4 and p65 leads to the inhibition of NF-κB signalling and may be a rational strategy to inhibit constitutive activation of NF-κB signalling in CRPC cells^48^^,^^49^. It was reported that BETi drugs induce the ubiquitination and degradation of p65^50^. We suppose that a similar mechanism may have contributed to the reduction of p65 levels observed in PC cells receiving the combination of GSK126 and JQ1.

Live-dead assay and Annexin V staining revealed that GSK126 and JQ1 combination overperformed the effects measured following single drug treatment. DU145 cells responded earlier and more to the combined treatment than PC3 cells. In these cells, the drugs combination triggered caspase 3/7 activation and induced PARP cleavage, committing cells to apoptosis following a G0/G1 cell cycle block. However, the effects of the combined treatment on caspases activation did not differ significantly from those observed following GSK126 single treatment, suggesting that, in these cells, also caspases independent mechanisms have contributed to the increase of apoptosis measured by mean of the Annexin V assay.

In PC3 cells, we found an increase of the G1 phase and the reduction of the S phase after 48 h of combined treatment. These data collectively suggest that the drug combination induces the cell cycle block at the G1 phase in the PC3 cell line. In these cells, we found a dramatic increase of p21 both following the combined treatment and JQ1 monotherapy which may have contributed on the one hand to the inhibition of cell proliferation and on the other hand to the inhibition of caspases activation through cell cycle arrest^51^.

Altogether, our data demonstrated that the combination of EZH2i and BETi is a rational pharmacological strategy to target c-myc in late stage mCRPC cells that are almost unresponsive to both GSK126 and JQ1 when used as a single drug treatment. From a clinical point of view our finding is relevant, considering that divergent AR (low) and MYC (high) transcriptional signatures predisposes patients to fail standard-of-care therapies and progress to the mCRPC stage^52^. Therefore, considering the heterogeneity of late stage mCRPC disease, appropriate patient selection through molecular diagnostics may be necessary to identify the right population that might benefit of the proposed myc-targeting strategy to maximise the therapeutic outcome.

Although the data presented in this manuscript are limited to *in vitro* models of advanced mCRPC and will require further validation in vivo, they constitute a rational base for further studies and for the development of more powerful bi-functional compounds.

## Supplementary Material

Supplemental Material
