# Supplementary figures and images for: Simultaneous administration of EZH2 and BET inhibitors inhibits proliferation and clonogenic ability of metastatic prostate cancer cells
Source: J Enzyme Inhib Med Chem. 2023 Jan 11;38(1):2163242. doi: 10.1080/14756366.2022.2163242 (PMC9848337; doi:10.1080/14756366.2022.2163242)

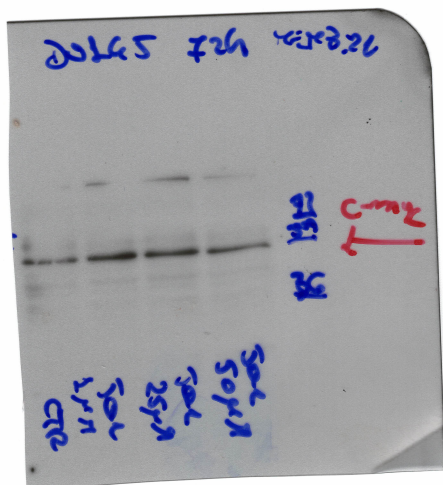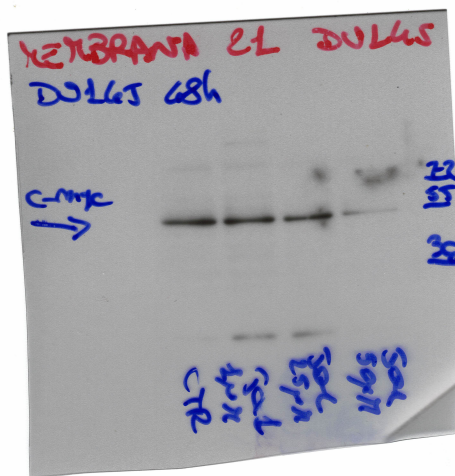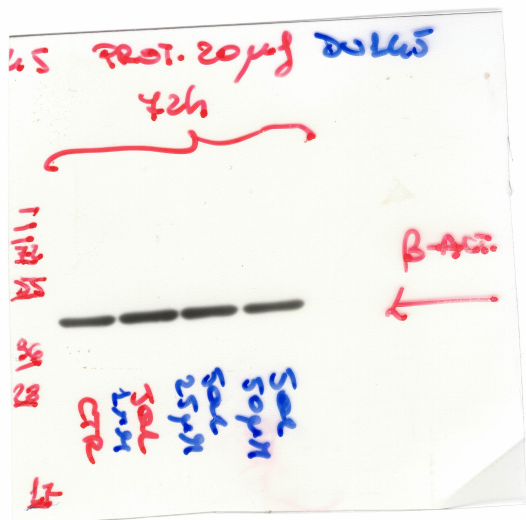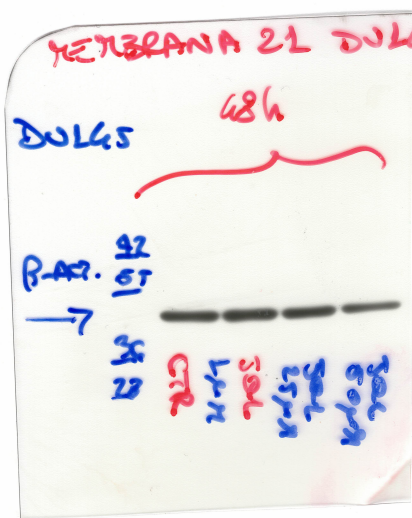

Supplement: Supplemental Material [file IENZ_A_2163242_SM4995.zip › Fig.S2 A-B.pdf]
