# Supplementary material for: Simultaneous administration of EZH2 and BET inhibitors inhibits proliferation and clonogenic ability of metastatic prostate cancer cells
Source: J Enzyme Inhib Med Chem. 2023 Jan 11;38(1):2163242. doi: 10.1080/14756366.2022.2163242 (PMC9848337; doi:10.1080/14756366.2022.2163242)
Supplement: Supplemental Material [file Fig.S2 C-D.pdf › Fig.S2 C-D.pdf]

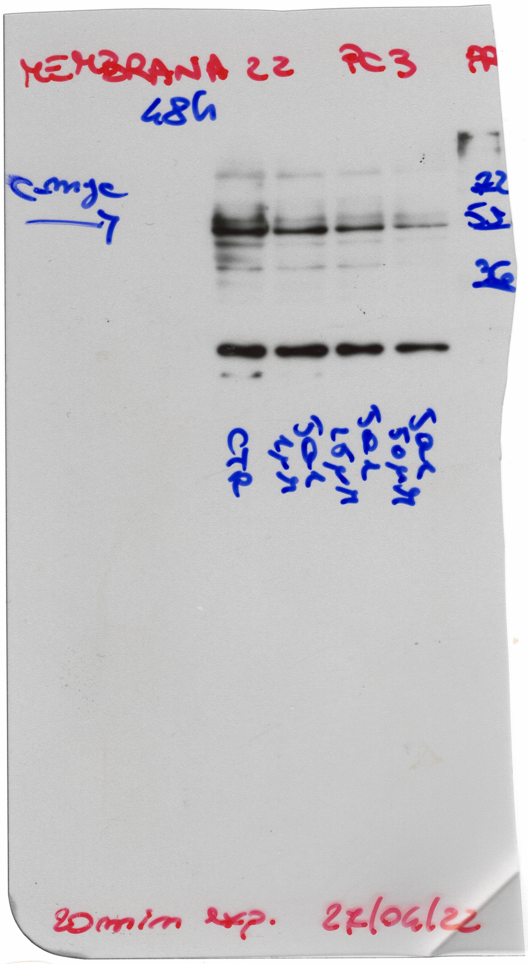
MEMBRANA 22 PC3
48h
C-myc
72
55
36
CTR
Sal 4µM
Sal 15µM
Sal 50µM
20min exp. 27/04/22

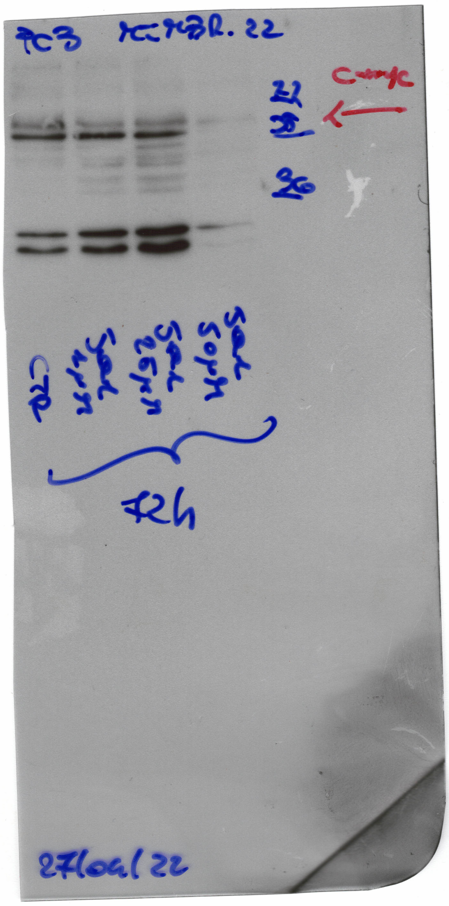
PC3 MEMBR. 22
C-myc
72
55
36
CTR
Sal 4µM
Sal 25µM
Sal 50µM
72h
27/04/22

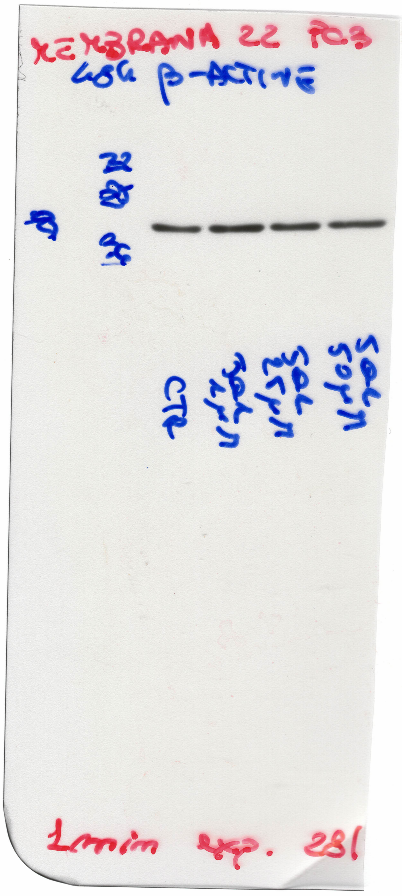
MEMBRANA 22 PC3
48h β-ACTINE
72
55
36
CTR
Sal 4µM
Sal 15µM
Sal 50µM
1min exp. 28/

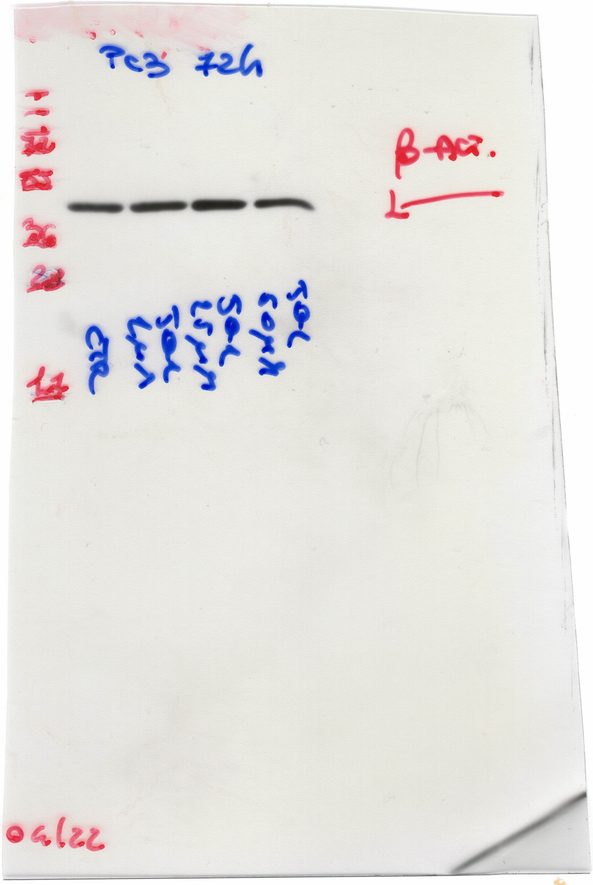
PC3 72h
β-ACT.
CTR
Sal 4µM
Sal 15µM
Sal 50µM
04/22
